# Genomancy: predicting tumour response to cancer therapy based on the oracle of genetics

**DOI:** 10.3747/co.v16i1.366

**Published:** 2009-01

**Authors:** P.D. Williams, J.K. Lee, D. Theodorescu

**Affiliations:** * Department of Public Health Sciences, Paul Mellon Urologic Cancer Institute, University of Virginia, Charlottesville, VA, U.S.A; † Department of Urology and Department of Molecular Physiology and Biological Physics, Paul Mellon Urologic Cancer Institute, University of Virginia, Charlottesville, VA, U.S.A

## Abstract

Cells are complex systems that regulate a multitude of biologic pathways involving a diverse array of molecules. Cancer can develop when these pathways become deregulated as a result of mutations in the genes coding for these proteins or of epigenetic changes that affect gene expression, or both^1^,^2^. The diversity and interconnectedness of these pathways and their molecular components implies that a variety of mutations may lead to tumorigenic cellular deregulation^3^–^6^. This variety, combined with the requirement to overcome multiple anticancer defence mechanisms^7^, contributes to the heterogeneous nature of cancer. Consequently, tumours with similar histology may vary in their underlying molecular circuitry^8^–^10^, with resultant differences in biologic behaviour, manifested in proliferation rate, invasiveness, metastatic potential, and unfortunately, response to cytotoxic therapy. Thus, cancer can be thought of as a family of related tumour subtypes, highlighting the need for individualized prediction both of disease progression and of treatment response, based on the molecular characteristics of the tumour.

## HIGH-THROUGHPUT TECHNIQUES AND APPROACHES

The development of high-throughput technologies that assess the expression of messenger rna (mrna) transcripts (“gene expression profiling”) has profoundly changed the fields of biology and medicine. By providing a snapshot of active cellular pathways, expression profiles can provide a more comprehensive picture of the biologic nature of a given tumour than can conventional clinical and pathologic indicators alone^11^.

These profiles have been put to progressively more sophisticated uses^12^–^14^. The discovery that expression profiles could be used to distinguish between different tumour types^15^ was followed by the discovery of subclasses of various cancers with distinct patterns of gene expression,^9^ some of which had implications for survival^8^,^10^,^16^. Those discoveries were closely followed by the development of prognostic prediction models, which have been used to predict disease progression or relapse in the absence of cytotoxic therapy^11^–^14^. The relative expression levels of a select number of mrna transcripts in these models can help to determine whether a patient should be treated. A good prognosis means that treatment can be avoided, and a poor prognosis suggests that further action is warranted. This approach minimizes the burden of treatment and maximizes the benefit to treated patients. As a demonstration of the utility of this technology, some of these models have been developed into diagnostic assays for use in the clinic^12^,^13^,^15^–^24^. Work is currently under way to generate similar predictors for tumour response to anti-neoplastic therapeutic regimens based on the gene expression signature of the individual tumour.

A classical approach to the development of these response predictors is to accrue patients in clinical trials, to profile the gene expression of individual tumours with microarrays, to discover biomarkers differentially expressed in patients responding and not responding to treatment, and then to generate prediction models that distinguish non-responders from responders. This approach is straightforward, but it suffers from several limitations, the most significant of which is that testing and monitoring patients undergoing each therapeutic regimen is extremely costly, slow, and limited to a very small number of current therapeutic options. Also, using this technique to generate successful prediction models for a particular drug, or its novel combinations, will be difficult, because trials using monotherapy and the numerous novel combinations are restrictive and reserved to phase i–ii designs. Predicting the efficacy of multiple drugs has been described^25^–^27^, but this approach suffers from the significant limitation that such models can be used only for the specific combinations—in effect preventing the combinatorial use of agents in other ways. This latter limitation is particularly relevant because many current clinical trials are evaluating novel anti-neoplastic drugs that could ultimately be used in combination with current agents.

Recent advances by our group and others have provided a potential solution to this problem. The National Cancer Institute (nci) has tested hundreds of thousands of potentially therapeutic compounds on a panel of 60 cell lines (nci–60)^28^ profiled using microarrays^29^. Sensitivity data, in the form of GI_50_ values (the concentration that inhibits the growth of the cell line by 50%), are publicly available for approximately 45,000 compounds. We^30,a^ and others^31^–^33^ have developed approaches to harness these data to generate predictions for the response of individual tumours to particular drugs.

## CO-EXPRESSION EXTRAPOLATION AND APPLICATION

Here, we describe our previously demonstrated method, the co-expression extrapolation (coxen) technique^30^, with its potential for the development of therapy response biomarkers without the limitations of the conventional approach described earlier. For each drug evaluated on the nci–60 assay, we can compare the expression patterns of sensitive and resistant cell lines to discover biomarkers and patterns of expression that correspond with drug sensitivity. For multiple reasons (such as inherent differences in environment and tissue type, and simple biologic variability), a gene may be differentially regulated in cell lines than in human tumours, and so we therefore determine which genes are concordantly expressed between the cell line panel and a set of human tumour microarray data. This step filters out uninformative or spurious genes. By focusing on the concordantly regulated genes, we can use a small number of biomarkers to make predictions. Using this method, we can also generate prediction models without intermingling data from training and test patient sets in any manner—a situation that should be avoided.^34^

Validation of this and other techniques is of key importance before any prediction model can be used clinically. Prospective clinical trials may be important for such validation, but extensive banks of formalin-fixed paraffin-embedded (ffpe) tissue samples may also serve as a key resource for retrospective validation of these models^35^, which if sufficiently robust and generalizable, may be sufficient for clinical use. Biomarker evaluation on prospectively collected tumour tissues from patients enrolled in clinical trials that have been completed are particularly valuable^19^. Because the coxen technique can generate prediction models requiring assessment of the expression of relatively few genes, quantitative polymerase chain reaction testing of ffpe tumour tissues can be used to determine a “predicted response” score, which can then be compared with the actual response of the patient to assess the accuracy of the predictions. Additionally, promising new technologies may enable high-throughput gene expression profiling of ffpe tissues themselves, facilitating assessment of the levels of many more genes^36^. It is also important to note that any predicted probabilities of response will be relative and not absolute; a patient with a higher predictive score will be more sensitive to a compound than one with a lower predictive score for the same compound. Therefore, during the process of validation, it will also be important to explore how prediction scores translate into real-world effects—namely, how differences in the coxen score translate into differences in patient outcomes and whether those differences are clinically significant.

Once validated models are developed, the coxen technique will have wide application. Validated models for several different treatment regimens used on a particular type of tumour can guide an oncologist toward selection of the optimal treatment for a specific patient. Validated models can also be used to predict response for tumours of particular tumour histology to U.S. Food and Drug Administration–approved drugs that have not previously been used to treat that particular tumour. This approach may prove very useful for patients with rare disease types or failure on established treatment regimens and for whom no clear guidelines exist for salvage regimens. These models can also be used to increase the likelihood that a novel drug will be found efficacious in clinical trials through the selective accrual of patients who are predicted to respond to the drug by virtue of analysis of their tumour.

Importantly, the coxen technique has shown some promise in drug discovery and thus may also be used in the future to prioritize drug leads: after screening newly synthesized drugs on cell-line panels, estimates can be made about the effectiveness of treatment. Another important application is the use of this technique for drug “repositioning” or “salvage,” which may offer significant new applications for agents that have already been studied in clinical trials, but whose target cancer populations may not have been optimally identified in the past.

However, much work remains to be done on the development and validation of these genomic drug response predictor models. Most chemotherapy regimens involve drugs administered in combinations, and therefore future work should devote particular attention to prediction of responses to these combination regimens. The individual and synergistic effects of the single compounds must also be understood so as to refine combinations for greater effectiveness and reduced toxicity. Furthermore, the large amount of biologic data outside the world of gene expression microarrays should also be integrated into these prediction models to further refine and improve prediction sensitivity and specificity. The combined application of these technologies and techniques may yet realize the promise of effective and individualized cancer therapy.
